# Keratolenticular adhesion removal for type 2 Peters anomaly: a case report

**DOI:** 10.1186/s40662-020-00203-5

**Published:** 2020-07-15

**Authors:** Zhangliang Li, Rui Zou, Yune Zhao

**Affiliations:** 1 School of Optometry and Ophthalmology, Wenzhou Medical University, Wenzhou, Zhejiang, China; 2 Key Laboratory of Vision Science, Ministry of Health P.R. China, Wenzhou, Zhejiang China

**Keywords:** Peters anomaly, Cataract, Endothelium, Pediatric

## Abstract

**Background:**

Type 2 Peters anomaly is a rare anterior segment disorder characterized by central corneal leukoma with keratolenticular adhesion and cataract. Performing cataract surgery without corneal tissue transplantation in patients of type 2 Peters anomaly is extremely rare and challenging. We present a case of type 2 Peters anomaly treated by peeling off the adhesion without penetrating keratoplasty (PKP), in which restoration of corneal transparency is observed.

**Case presentation:**

An 11-month-old female infant of type 2 Peters anomaly presented with bilateral corneal opacity with distinct demarcation, keratolenticular adhesion and cataract, which was first noted at the age of 3 months. By peeling off the adhesion from corneal endothelium combined with lensectomy and vitrectomy, there was a gradual reduction in corneal opacity and improvement in visual acuity after surgery over a 2-year period. Her visual acuity had improved from light perception preoperatively to 20/50 at the latest follow-up. No sight-threatening postoperative complications were noted.

**Conclusion:**

It is safe and effective to peel off the keratolenticular adhesion in patients of type 2 Peters anomaly presented with distinctly demarcated corneal opacity.

## Background

Peters anomaly is a rare form of congenital anterior segment dysgenesis characterized by central corneal opacity with defects in the posterior stroma, Descemet’s membrane, and endothelium [1]. Corneal leukoma with iridocorneal adhesion is classified as type 1 Peters anomaly, and corneal leukoma with cataract and keratolenticular adhesion is classified as type 2 Peters anomaly [2]. Type 2 anomaly is usually associated with poorer visual outcomes compared with type 1 anomaly [3, 4].

Previous management options for neonatal corneal opacities include mydriatics, occlusion therapy, peripheral iridectomies, and penetrating keratoplasty (PKP) [3]. The prognosis for PKP in children with Peters anomaly type 1 can be excellent, with a graft success rate of 53% to 90% [2, 3]. However, the presence of keratolenticular adhesion and cataract in type 2 anomaly necessitates lensectomy and vitrectomy, which aggravates graft survival after PKP [5]. Patients with type 2 anomaly have a lower success rate than those with type 1 anomaly [2]. Cataract surgical techniques without concurrent PKP has become an important option for type 2 Peters anomaly [6, 7]. Herein, we report a case of bilateral type 2 Peters anomaly successfully treated with peeling off the adhesion between the capsular bag and corneal endothelium concurrent with lensectomy and anterior vitrectomy. The cornea restored and maintained excellent transparency and an excellent visual outcome was achieved at the two-year follow-up after surgery without need for further corneal tissue transplantation.

## Case presentation

**Fig. 1:**
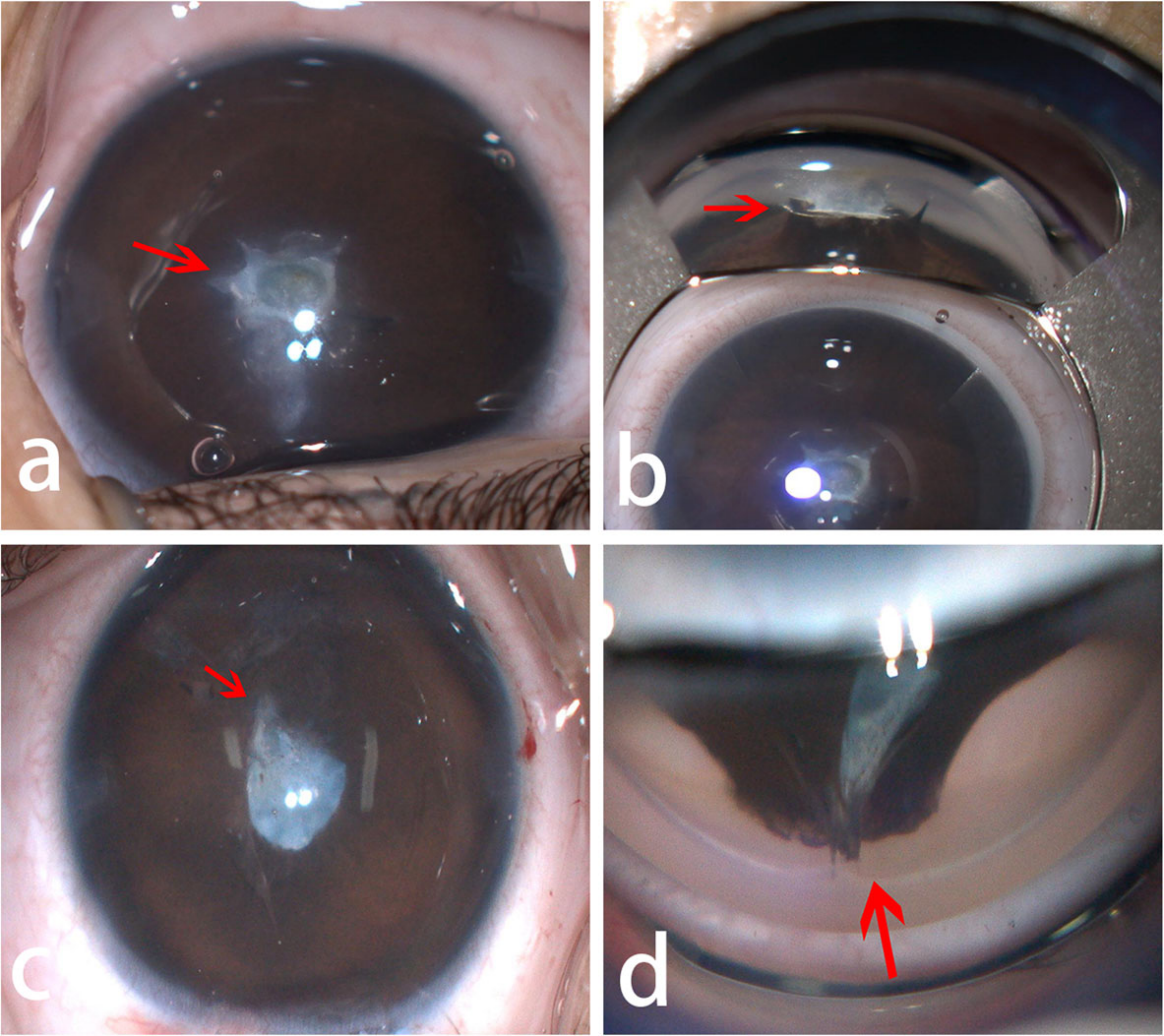
Preoperative photos of anterior segment. **a** The anterior segment of the patient’s right eye; **b** View of gonioscope of the patient’s right eye; **c** Anterior segment of the patient’s left eye; **d** View of gonioscope of the patient’s left eye (arrowheads shows area of adhesion)

An 11-month-old female infant presented with bilateral corneal opacity, which was first noted at the age of 3 months. The patient was born full term with an uneventful birth history, and no history of maternal infection or family history of ophthalmologic disease. Ophthalmic examination revealed central corneal opacity approximately 3.0 mm in diameter in the right eye and 2.0 mm in diameter in the left eye, with an underlying keratolenticular adhesion and a cloudy cataract, and that the patient could not trace the light (Fig. 1). Contact ultrasound A-scans revealed axial lengths of 18.28 mm and 18.39 mm in the right and left eyes, respectively. Intraocular pressure (IOP) were 14 mmHg in both eyes, measured by a handheld tonometer (Icare Finland Oy Vantaa, Finland). There were no systemic anomalies.

The patient’s parents hesitated to accept PKP and fully understood the risks of performing PKP in infants because they had consulted several surgeons before coming to our clinic to seek a second opinion. Her parents were subsequently offered an option of adhesiolysis and adhesive membrane removal combined with lensectomy and vitrectomy. Surgeries were performed at the age of 11 months on 17th and 19th October 2017 in the right and left eye, respectively.

### Surgical technique

Surgeries were performed by an experienced surgeon (Y.E.Z.) under general anesthesia using the Accurus with the venturi vacuum system (Alcon Laboratories, Inc.); the cut rate was 2000 per minute and vacuum was 350 mmHg. A corneoscleral incision was made superiorly and four 1.0 mm paracentesis were created in each quadrant. The anterior chamber was initially filled with ophthalmic viscosurgical device (OVD), and the neck of the keratolenticular adhesion was cut using intraocular scissors. There was comprehensive iris posterior synechia. The pupillary aperture was enlarged by four iris hooks through paracentesis in each quadrant after adhesiolysis. Then, a partially resorbed lens and peripheral anterior capsule contraction with zonular elongation were noted underneath the keratolenticular adhesion. The anterior capsular defect was extended to an anterior capsulorhexis of approximately 5.0 mm diameter using a 23-gauge vitrector, while the anterior chamber was maintained by a 23-gauge infusion cannula. After the mode was switched to irrigation/aspiration, the cortex was carefully aspirated. Next, a posterior capsulotomy with a 3.0 mm diameter was performed with the vitrector and the anterior part of the vitreous volume was removed using the same vitrectomy settings. Before the end of the procedure, the residual adhesion was gently peeled off by capsulorhexis forceps curvilinearly following the demarcation line. Surgery was concluded with reformation of the anterior chamber with balanced salt solution and closure of the corneoscleral incision with 10–0 nylon sutures, leaving both eyes aphakic (Additional file 1). No unexpected intraoperative complications were encountered. Clinical manifestations were similar in both eyes.

**Additional file 1.** A surgical video of right eye manifests the major procedures

**Fig. 2:**
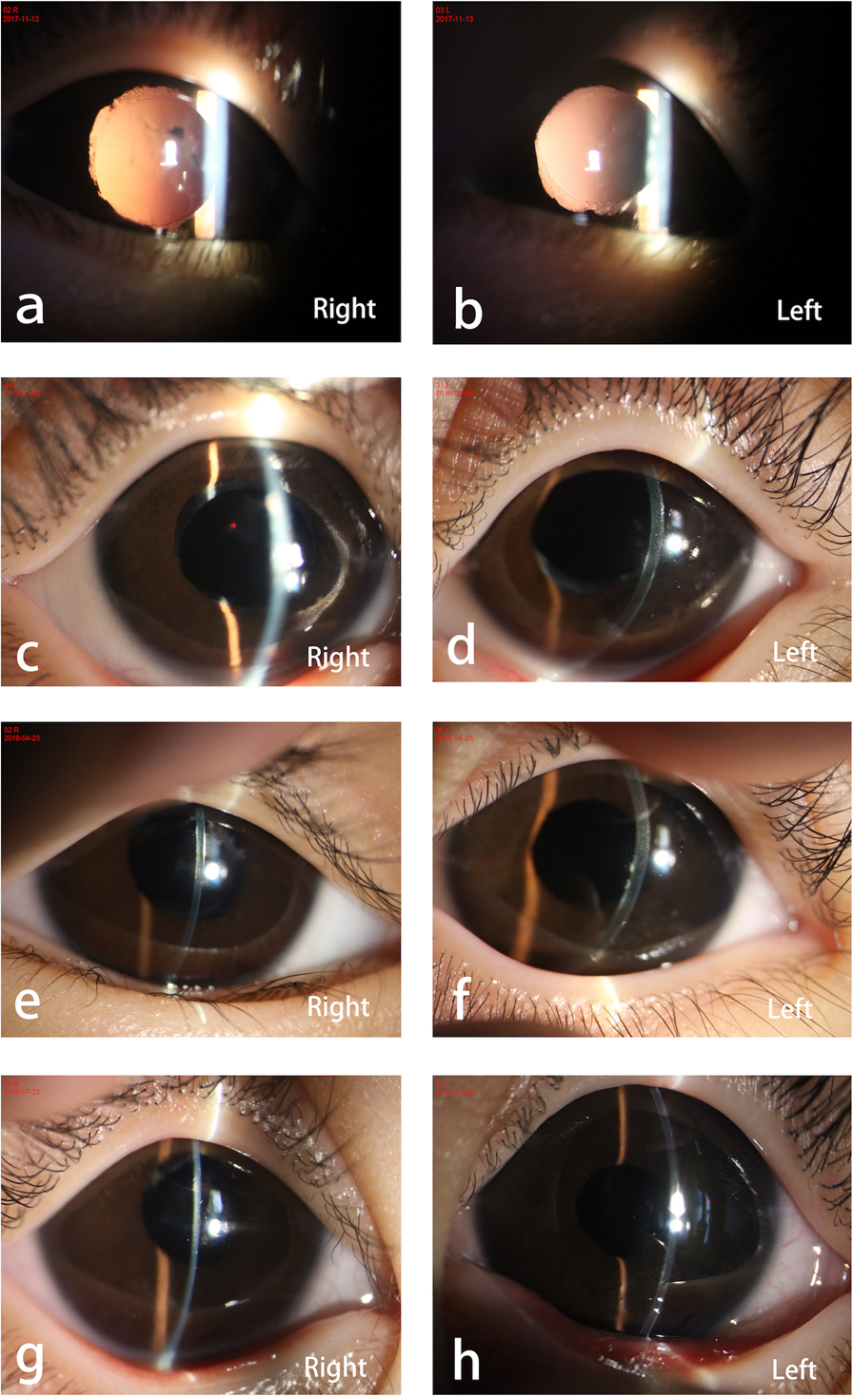
Photo montage shows central corneal opacity being gradually reduced in both right and left eyes. (**a** & **b**) 1 month after surgery; (**c** & **d**) 3 months after surgery; (**e** & **f**) 6 months after surgery; (**g** & **h**) 9 months after surgery

**Fig. 3:**
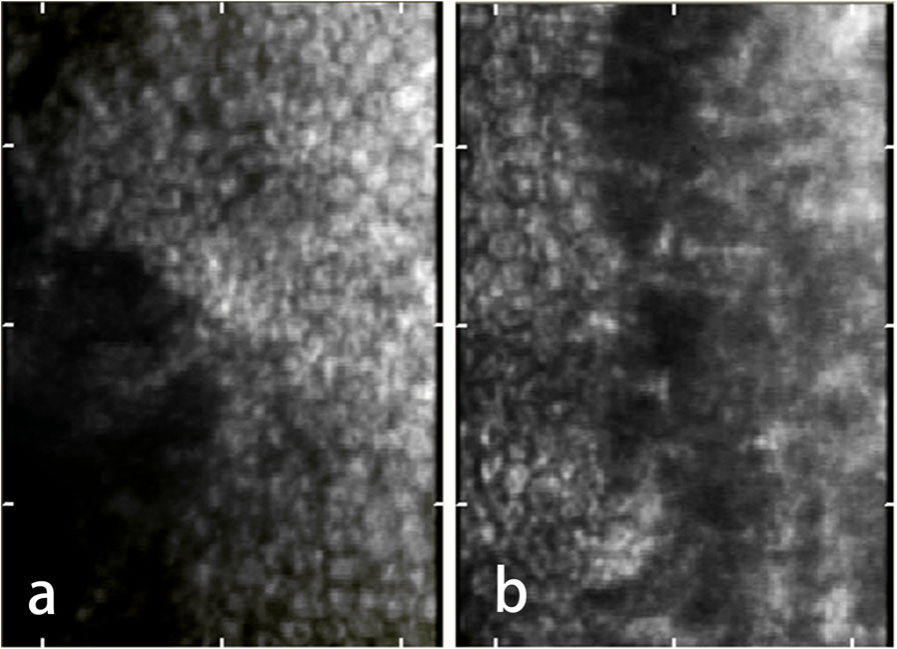
Screenshots of the noncontact specular microscopy measuring the center of cornea at one year after surgery. **a** Right eye; **b** Left eye

Topical treatment consisted of steroidal eye drops gradually tapering over 4 weeks, antibiotic eye drops 4 times daily for 2 weeks, and mydriatic eye drops (phenylephrine hydrochloride and tropicamide compound) once a day for 4 weeks. A gradual reduction in central corneal opacity and improvement in the visual acuity (VA) was noted (Fig. 2). Non-contact specular microscopy at one year postoperatively showed large heteromorphic endothelial cells at the borderline between the normal endothelium and the central defect (Fig. 3). At the latest follow-up that was two years after surgery, the corneal had excellent transparency, with corrected Teller VA of 20/50 in both eyes and IOP of 15 mmHg in the right eye and 14 mmHg in the left eye. There was no evidence of glaucoma.

## Discussion and conclusions

Cataract surgery without penetrating keratoplasty in type 2 Peters anomaly is very rare and challenging due to poor visualization through corneal opacity, and that the presence of iridocorneal and keratolenticular adhesion could lead to small pupillary aperture and anterior capsule rupture. Therefore, there are limited cases on performing cataract surgery without penetrating keratoplasty in type 2 Peters anomaly. Here, we report a case of type 2 Peters anomaly that has successfully gained excellent corneal transparency and visual outcome after peeling off the adhesion from the cornea and lensectomy, with concurrent anterior vitrectomy and post-op hyperopia correction.

Soh and colleagues described a novel surgical technique for type 1 Peters anomaly; using a custom-made silicone soft-tip probe to debride the unhealthy endothelium (“Endothelial Scraping”) while preserving Descemet’s membrane [8]. They confirmed resolution of the endothelial defect after endothelial scraping by the absence of trypan blue uptake at the posterior corneal surface. In our case, we peeled off the adhesive tissue from the endothelium by capsulorhexis forceps following the demarcation line, in order to break the contact inhibition of cell migration at the ridge formed by demarcation line. Similarly, we observed endothelial cell migration at the borderline by non-contact specular microscopy. In an ex vivo tissue culture experiment on cadaveric human corneas, complete endothelial recovery occurred through centripetal cell migration after intentional damage to the endothelium [9], which strongly relies on the presence of healthy endothelial cells nearby. It could be argued that the restored corneal transparency is attributed to the fact that the keratolenticular adhesion is due to late apposition rather than failure of separation. There is no way to confirm this since we did not perform a ultrasound biomicroscopy before surgery, which may help distinguish late apposition from failed separation [10]. Nevertheless, when peeling off the adhesion, the strong strength of adhesion implies it more likely to be failure of separation. The child in our case was examined elsewhere and then referred to our clinic at the age of 11 months. A better prognosis could be expected if the surgery had been performed earlier.

Medsinge and colleagues removed cataract while the adhesion of capsular bag to the cornea was left intact in type 2 Peters anomaly [6]. They believe that peeling the adhesion could further damage the adjacent endothelial cells and lead to enlargement of corneal opacity. However, there is an obvious demarcation in our case that represents the border of adhesion and weakens the shearing force when peeling the adhesion. It turns out that the corneal opacity was not enlarged by our procedure.

Hou and colleagues utilized an image-guided femtosecond laser platform to perform an anterior capsulotomy in type 2 Peters anomaly in which the peripheral cornea remained clear [7]. Indeed, femtosecond laser-assisted cataract surgery is a safe and effective choice in cases of type 2 Peters anomaly, but the high cost of the machine is a limiting factor.

In summary, the surgical indication of our procedures is limited to type 2 Peters anomaly cases of limited central corneal opacity with a distinct demarcation line. PKP should still be the surgical choice for patients with extensive keratolenticular adhesion or indistinct demarcation of corneal opacity. Peeling off the keratolenticular adhesion in patients of type 2 Peters anomaly presenting with distinctly demarcated corneal opacity could be safe and effective with long-term outcome monitoring and careful patient selection.

## Data Availability

All data generated or analyzed during this study are included in this published article [and its supplementary information files].
